# Exploring Types of Photonic Neural Networks for Imaging and Computing—A Review

**DOI:** 10.3390/nano14080697

**Published:** 2024-04-17

**Authors:** Svetlana N. Khonina, Nikolay L. Kazanskiy, Roman V. Skidanov, Muhammad A. Butt

**Affiliations:** Samara National Research University, 443086 Samara, Russiakazanskiy@ipsiras.ru (N.L.K.);

**Keywords:** photonic neural networks, artificial intelligence, spiking neural network, feedforward neural network, recurrent neural network

## Abstract

Photonic neural networks (PNNs), utilizing light-based technologies, show immense potential in artificial intelligence (AI) and computing. Compared to traditional electronic neural networks, they offer faster processing speeds, lower energy usage, and improved parallelism. Leveraging light’s properties for information processing could revolutionize diverse applications, including complex calculations and advanced machine learning (ML). Furthermore, these networks could address scalability and efficiency challenges in large-scale AI systems, potentially reshaping the future of computing and AI research. In this comprehensive review, we provide current, cutting-edge insights into diverse types of PNNs crafted for both imaging and computing purposes. Additionally, we delve into the intricate challenges they encounter during implementation, while also illuminating the promising perspectives they introduce to the field.

## 1. Introduction

Photonic neural networks (PNNs) mark a pioneering approach to neural computing, exploiting the velocity and concurrency of light to enhance information processing efficiency [1,2,3]. By capitalizing on optical components and principles, PNNs present compelling remedies to long-standing impediments in traditional electronic neural networks, such as speed constraints and energy consumption [2,4]. PNNs embrace a spectrum of architectures, spanning feedforward, recurrent, convolutional, and spiking neural networks, each meticulously crafted for distinct tasks and domains [5,6]. Noteworthy advantages of PNNs encompass their capacity for lightning-fast computation, vast parallelism, and innate adaptability to certain data processing challenges like image recognition and optimization tasks [7,8]. Furthermore, PNNs demonstrate potential in surmounting emerging hurdles in artificial intelligence (AI), photonics, and information processing, heralding a new era of computing paradigms poised to revolutionize an array of fields, from healthcare to telecommunications [9,10].

Quantum neural networks (QNNs) and PNNs represent two distinct paradigms in advanced computing, each employing unique principles for data processing and analysis [11]. QNNs leverage quantum mechanics principles like superposition and entanglement, whereas PNNs utilize photonics for neural network operations. The primary distinction lies in their hardware platforms; QNNs rely on quantum processors manipulating qubits, while PNNs use photonic devices. Regarding scalability, QNNs face challenges related to qubit coherence times and error rates, whereas PNNs encounter obstacles in fabricating precise photonic components and integrating them with electronic infrastructure. Additionally, QNNs show potential for exponential speedup in tasks such as optimization and cryptography due to quantum parallelism and annealing, while PNNs may excel in applications requiring low latency and high bandwidth, like telecommunications and data processing [12]. Despite their differences, both QNNs and PNNs hold promise for advancing machine learning and computing, contingent upon specific task requirements and underlying hardware capabilities. In our view, photonics stands out for its exceptional capabilities in interconnects and communications, particularly due to its high bandwidth potential, effectively addressing the trade-offs associated with bandwidth and interconnectivity [13,14,15]. Decades ago, the benefits of photonics for neural networks were predictable, with pioneering work led by Psaltis and others, who introduced spatial multiplexing methods, permitting comprehensive all-to-all interconnection [16]. However, practical applications of PNNs faced obstacles due to limitations in low-level photonic integration and packaging technologies at that time. Nonetheless, the landscape of PNNs has seen significant changes with the advent of large-scale photonic assembly and integration methods [17,18]. For example, silicon photonics has become a leading platform for producing extensive and cost-effective optical systems [19,20,21]. Concurrently, various evolving applications, such as resolving nonlinear optimization problems and processing multichannel GHz analog signals in real time, are seeking innovative computing platforms to fulfill their computational needs [22]. These advancements illuminated fresh opportunities and pathways for advancing PNNs [23].

Particularly compelling is their application in deep learning and pattern recognition, where PNNs harness the parallel processing capabilities of light to execute intricate neural network operations at remarkable speeds, facilitating swift inference and training tasks that strain conventional electronic systems [24,25,26]. Moreover, PNNs boast exceptional energy efficiency owing to the minimal losses inherent in photonics, rendering them well-suited for deployment in energy-constrained settings and portable devices. In addition, PNNs show promise in optical computing, where they excel in tasks like image processing, cryptography, and optimization with unmatched efficiency [27,28,29,30]. In the realm of telecommunications and data processing, PNNs stand to revolutionize optical signal processing, enabling rapid data transmission and processing for cutting-edge communication networks. Furthermore, PNNs hold significant potential in advancing biomedical imaging and sensing technologies, enabling the real-time analysis of biological data with precision and sensitivity. Overall, the versatility and significance of PNNs highlight their capacity to propel innovation across diverse fields, offering unprecedented speed, efficiency, and scalability for the next generation of computing and information processing systems. PNNs leverage optical technologies to perform certain aspects of neural network computation, offering potential benefits in terms of speed, energy efficacy, and parallelism. Several types of PNNs were proposed and studied [2], which are discussed in Section 2. The prospects and challenges of the PNNs are briefly discussed in Section 3, and the paper ends with a brief discussion and concluding remarks. 

The promising potential of PNNs is hindered in real-world usage cases for several reasons. Firstly, the complexity and cost associated with fabricating photonic components capable of performing neural network operations limited their widespread adoption. Photonic devices require precise manufacturing processes and sophisticated materials, resulting in high production costs and scalability limitations. Additionally, challenges arose in integrating photonic components with existing electronic infrastructure due to compatibility and interoperability issues. Furthermore, the efficient implementation of PNNs has been impeded by the lack of standardized design methodologies and optimization algorithms tailored specifically for them. Concerted efforts across multiple domains are necessary to make PNNs a realistic prospect for real-world adoption. Technological advancements in materials science and fabrication techniques could reduce manufacturing costs and enhance the performance of photonic devices. Moreover, crucial research efforts are needed to develop standardized design frameworks, optimization algorithms, and integration strategies tailored for PNNs. Collaborations among academia, industry, and government bodies to invest in research and development initiatives can accelerate progress in these areas. Additionally, educational programs aimed at fostering interdisciplinary expertise bridging photonics and machine learning domains would cultivate a skilled workforce capable of driving innovation in PNN technology. 

## 2. Types of PNNs

**Figure 1:**
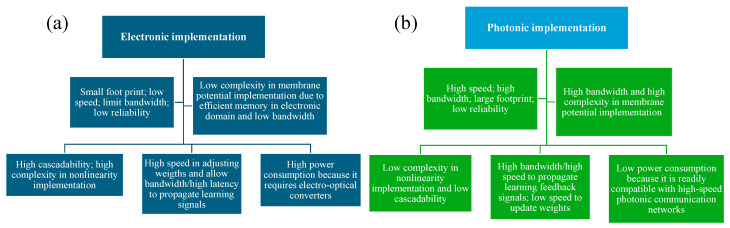
Electronic versus photonic implementation of neuron functions. (**a**) Electronic implementation, (**b**) Photonic implementation.

PNNs represent a paradigm shift in computing by harnessing light’s inherent advantages over traditional electronic systems. This emerging technology promises breakthroughs in speed, energy efficiency, and scalability, crucial for addressing the escalating demands of data-intensive tasks like ML and AI. When assessing the outcomes of processing speed, energy consumption, and accuracy between PNNs and traditional electronic neural networks, notable advantages emerge in specific domains [2]. PNNs exhibit remarkable processing speed, owing to the inherent parallelism ingrained in optical computing, facilitating simultaneous data processing across numerous channels. Empirical evidence from various studies underscores processing speeds several orders of magnitude faster than those achieved by electronic counterparts. Additionally, PNNs boast lower energy consumption per computation, owing to the fundamental properties of light propagation, leading to diminished heat dissipation and power consumption in contrast to electronic devices [31,32]. Nonetheless, despite excelling in processing speed and energy efficiency, potential trade-offs regarding accuracy may arise. While PNNs demonstrate promising outcomes in tasks such as pattern recognition and image processing, their accuracy may fluctuate based on the neural network architecture’s complexity and the precision of optical components employed [30]. Comparative analyses between PNNs and electronic neural networks elucidate these trade-offs, delineating areas where PNNs excel, as well as other areas necessitating further optimization to attain comparable accuracy levels [33]. Figure 1 presents a comprehensive overview contrasting the functionalities of photonic and electronic implementations of neurons.

**Figure 2:**
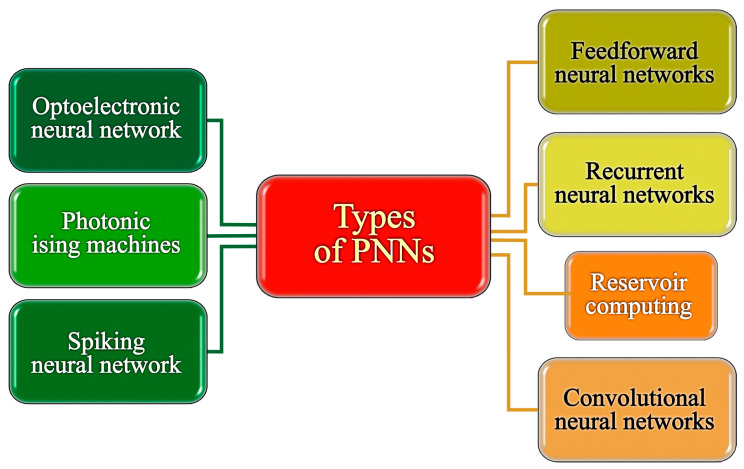
Types of PNNs.

In this section, we delved into several pivotal types of PNNs that stand as focal points in imaging and computing research, illuminating their significance and widespread exploration in the field, as presented in Figure 2. Feedforward neural networks (FNNs) provide a foundational framework for pattern recognition and classification tasks by mapping input data to output predictions through layers of interconnected neurons [34]. Recurrent neural networks (RNNs) host the crucial concept of feedback loops, enabling them to process sequential data with temporal dependencies, making them essential for tasks, for instance, natural language processing and time series analysis [35]. Convolutional neural networks (CNNs) excel in image and video processing tasks, leveraging shared weights and local connectivity to derive hierarchical features, making them indispensable in computer vision applications [36,37]. Reservoir computing (RC), a subset of recurrent networks, offers advantages in processing temporal data efficiently, particularly in tasks where memory and context play vital roles [19]. Spiking neural networks (SNNs), stimulated by biological neurons’ spiking behavior, offer low-power neuromorphic computing capabilities appropriate for brain-inspired computing tasks and efficient event-based processing [19]. Photonic Ising machines (PIMs) exploit principles from statistical physics to solve optimization problems efficiently, while optoelectronic neural networks (ONNs) leverage light-based communication for high-speed, parallel processing, offering promising solutions for large-scale computational tasks [19]. In our opinion, each of these architectures brings its own set of strengths to the table in the realm of computing and imaging. Together, they push the boundaries of AI forward, opening new possibilities and paving the way for tackling a wide range of real-world problems.

Photonics offers a multitude of advantages across various types of neural networks. In FNNs, photonics enables high-speed processing due to the intrinsic speed of light, enhancing computational efficiency. The parallel nature of photonics allows for the simultaneous processing of multiple inputs, enhancing the network’s throughput. In RNNs, photonics facilitates the efficient handling of time-varying signals, which is crucial for temporal processing tasks. Additionally, the inherent parallelism of photonics can accelerate computations in CNNs, which excel in tasks involving spatial relationships. Photonics is also well-suited for RC, where its high bandwidth and low latency enable rapid information processing. SNNs benefit from photonics’ ability to efficiently transmit and process sparse, asynchronous signals akin to biological neurons. PIMs exploit photonics’ parallelism for solving optimization problems efficiently. Lastly, in ONNs, photonics seamlessly integrates with electronics, offering low-latency communication and high-bandwidth connections, thus improving network performance. Overall, photonics presents a promising avenue for enhancing the speed, efficiency, and performance of diverse neural network architectures.

Considering the distinct characteristics of different types of PNN described earlier, we classified them into two primary groups. On the right side of Figure 2, we assembled a category corresponding to traditional artificial neural networks (ANNs) that could potentially be realized using optical or photonic technologies. Conversely, architectures tailored specifically to exploit the unique properties of PNN are positioned on the left side of Figure 2. Subsequent subsections will delve into each of these categories in greater depth.

### 2.1. Feedforward Neural Networks (FNNs)

**Figure 3:**
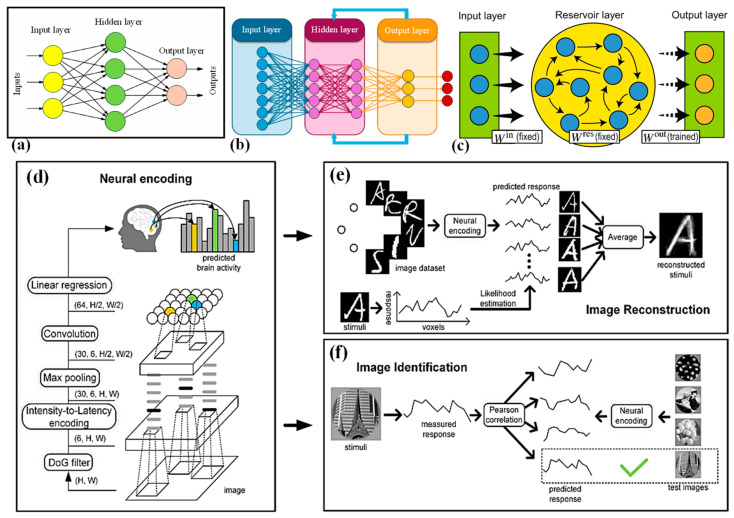
Structure of (**a**) FNN, (**b**) RNN, (**c**) typical RC [53]. (**d**) The depiction of the encoding prototype. The model employs a 2-layer SCNN to derive graphic topographies from input pictures and utilizes linear regression models to forecast the fMRI responses for each voxel. (**e**) The schematic for the image-rebuilding endeavor, targeting the reconstruction of perceived images from brain activity. (**f**) An illustration for the image identification chore, focused on discerning the perceived image based on fMRI responses [67,69,70].

FNNs represent a fundamental architecture in ANNs, categorized by the unidirectional flow of information from input nodes through one or more hidden layers to output nodes, as reported in Figure 3a [38]. In essence, FNNs process input data by passing them through a series of interconnected layers of neurons, each layer transforming the data representation to derive increasingly abstract features [39]. These networks are trained using approaches such as backpropagation, where the inconsistency between the projected output and the actual target is minimized through iterative adjustments to the network’s parameters. FNNs find widespread applications in countless spheres, containing image and speech recognition, natural language processing, and financial forecasting, owing to their capacity to learn multifaceted patterns and associations in data [40]. Despite their simplicity compared to more complex architectures, FNNs serve as foundational models upon which more advanced network designs are built, making them a cornerstone of modern ML and AI [41].

Differential equations manifest across numerous domains of science and engineering, offering a valuable means of describing various physical phenomena. They typically manifest as initial or boundary value problems, wherein conditions at the inception of a process or boundary points are stipulated to yield an explicit solution. Employing numerical methods [42], such as finite difference methods, serves as a useful strategy for approximating these equations. Furthermore, neural networks appeared as a viable instrument for this purpose [43,44]. The realm of neural network architectures boasts a vast array of possibilities. Notably, FNNs demonstrated utility in solving differential equations, as evidenced by seminal works in the field [45]. Within the framework of FNNs, two specific approaches (trial solution method and modified trial solution method) [46,47] have gathered substantial attention in the literature over recent decades, displaying substantial promise.

FNNs present a potential method for resolving differential equations. Nevertheless, the consistency and precision of the approximation still pose unresolved challenges within the existing literature. Computational methodologies are generally heavily reliant on various computational parameters and the selection of optimization techniques, which must be considered in conjunction with the structure of the cost function. In [48], the resolution of a straightforward yet pivotal stiff ordinary differential equation representing a damped system is proposed. Two computational strategies are proposed for resolving differential equations using neural forms: the conventional but still relevant approach of trial solutions defining the cost function and a recent direct formulation of the cost function associated with the trial solution process. It is worth noting that these configurations can be readily extended to encompass the solution of partial differential equations. Through an exhaustive computational analysis, the potential to discern preferable choices for parameters and methodologies is demonstrated. Additionally, light was shed on intriguing phenomena observable in the simulations of neural networks. 

### 2.2. Recurrent Neural Networks (RNNs)

RNNs characterize a specialized class of ANNs engineered to manage sequential data [49,50]. They achieve this by incorporating connections that create directed cycles within the network graph, facilitating dynamic temporal behavior and the processing of sequences of variable lengths. Diverging from the linear flow of information characteristic of FNNs, RNNs feature connections that loop back, empowering them to retain and exploit information from past states. The assembly of RNN is depicted in Figure 3b. 

This unique trait renders RNNs particularly adept at tasks revolving around sequential data, including but not limited to time series prediction, natural language processing, speech recognition, and music generation. Notably, RNNs serve crucial roles in language modeling and text generation, exemplified in machine translation systems where they encode a source sentence into a fixed-length vector depiction before decoding it into a target sentence, enabling seamless translation across languages. Similarly, in sentiment analysis within natural language processing, RNNs excel at discerning sentiment in a text by analyzing the context of individual words within the broader sentence context. Moreover, RNNs contribute significantly to speech recognition systems by effectively modeling the temporal dependencies present in audio data, thereby accurately transcribing spoken words. In summary, recurrent neural networks hold pivotal importance in managing sequential data across diverse domains, owing to their proficiency in capturing temporal dependencies and processing sequential information with precision.

RNNs offer powerful capabilities for sequential data processing, but they come with several challenges and limitations. One significant challenge is the vanishing gradient problem, where the gradients diminish exponentially as they propagate backward in time during training, hindering long-range dependencies learning. Additionally, RNNs struggle with capturing long-term dependencies due to their inherent sequential nature, making it difficult to retain information over extended sequences. Moreover, training RNNs can be computationally expensive and time-consuming, especially with large datasets. Another limitation is their exertion in managing variable-length sequences efficiently, as they necessitate fixed-length input and output vectors. Lastly, RNNs are prone to overfitting, particularly when addressing noisy or sparse data, necessitating careful regularization techniques to mitigate this issue. Despite these challenges, advancements like Long Short-Term Memory [51] and Gated Recurrent Unit architectures [52] were established to alleviate some of these constraints and improve the effectiveness of RNNs in various tasks.

### 2.3. Reservoir Computing (RC)

RC is a cutting-edge archetype in the field of ML, particularly within the domain of RNNs. Unlike traditional RNNs, where the recurrent connections are subject to training, RC employs a fixed, randomly generated recurrent network called the “reservoir.” This reservoir acts as a dynamic memory system that preserves temporal information and captures complex temporal dependencies within sequential data, as shown in Figure 3c. The input signals are injected into the reservoir, where they undergo nonlinear transformations, leading to rich representations of the input data. The key revolution of RC lies in the separation of the training phase from the reservoir dynamics, allowing for simpler and more efficient learning algorithms. During the training phase, only the output weights are adjusted utilizing simple linear regression or other optimization techniques, enabling rapid training and efficient adaptation to various tasks. RC has shown remarkable performance across a range of applications, including time series prediction, speech recognition, natural language processing, and robotics, making it a promising approach for addressing complex temporal problems in both research and practical applications. Its flexibility, simplicity, and superior performance propelled it to the forefront of modern ML methodologies, fostering ongoing research and development in the field.

In the work presented by Sakemi et al. [53], a novel technique aimed at diminishing the reservoir’s size by incorporating either past states or evolving dynamics directly into the output layer at the current time step was proposed. To shed light on the underlying principle of model size reduction, a thorough analysis is conducted leveraging the data processing capability framework proposed by Dambre et al. [54]. Furthermore, the efficacy of these techniques was assessed through rigorous evaluations of time-series forecast tasks, including the general Hénon-map and NARMA. Remarkably, these findings demonstrate that the anticipated approaches can achieve a reduction in reservoir size of up to one-tenth without significantly amplifying the regression error.

RC presents a promising approach to sequential data processing, yet it also confronts several challenges and limitations [55]. One significant challenge is the model and optimization of the reservoir itself, as finding the right architecture and parameters can be highly task-dependent and nontrivial. Additionally, training the readout layer to effectively derive information from the reservoir states requires careful tuning and regularization to prevent overfitting, especially in the presence of noisy or high-dimensional data. Furthermore, RC systems may struggle with capturing long-term dependencies in sequential data, particularly in cases where the underlying dynamics are highly complex or chaotic. Moreover, scalability can be an issue with large-scale reservoirs, as the computational and memory requirements grow proportionally with the size of the reservoir, potentially limiting its applicability to real-world problems [56]. Despite these challenges, ongoing research aims to address these limitations and further enhance the capabilities of RC for a wide range of tasks and applications [57].

### 2.4. Convolutional Neural Networks (CNNs)

CNNs are a cornerstone in the realm of AI, predominantly in computer vision tasks. These networks are inspired by the biological visual cortex’s structure and operation, leveraging layers of interconnected neurons to process visual information [58]. At the core of CNNs lies the convolution operation, where filters or kernels are utilized to input data to extract meaningful features [59]. Through a process of convolution, nonlinear activation, pooling, and often repeated layers, CNNs can effectively learn hierarchical representations of features from raw input data. This hierarchical learning enables CNNs to automatically learn and identify patterns, textures, and shapes within images, making them exceptionally powerful for tasks such as image classification, object detection, and image segmentation. CNNs demonstrated extraordinary performance across various domains, including healthcare, autonomous vehicles, and satellite imagery scrutiny, continually pushing the boundaries of what is possible in computer vision and pattern recognition [60].

One of the pivotal aspects of a CNN model lies in its ability to generalize effectively to unseen data. Overfitting stands out as a prevalent issue within CNN networks, manifesting when the model fits the training dataset well but struggles to generalize to new examples outside its training scope [61]. This phenomenon occurs due to the model memorizing training examples without truly learning from them. Mitigating overfitting entails strategies such as expanding the training dataset, employing data augmentation methods, simplifying architecture, applying regularization methods, and implementing early stopping mechanisms. Furthermore, other challenges in training a CNN model include the occurrence of exploding gradients and class imbalances. Exploding gradients become apparent when the training model fails to learn from the data after a certain number of epochs, resulting in overflow and NaN loss values for the error gradient. This instability in learning can be addressed through measures like redesigning the network architecture, gradient clipping, and selecting suitable activation functions. Class imbalance represents another hurdle, characterized by a significant nonuniform distribution of specimen classes [62]. Addressing this issue during model training has long been a substantial challenge in ML.

Recently, CNNs have gathered noteworthy consideration for their inspiring advancements in computer vision. Many research endeavors employed comparative parallel analysis to juxtapose the divergence patterns of CNN and functional magnetic resonance imaging (fMRI) representations [63,64]. These explorations revealed similarities, suggesting that the human visual cortex shares hierarchical depictions akin to CNNs. Consequently, CNN-built encoding models gained widespread acceptance and verified exceptional performance [65,66]. However, it is vital to recognize that despite the triumph of CNNs in encoding tasks, the differences in how CNNs and the brain process visual data should not go unnoticed. Precisely predicting brain responses to numerous stimuli remains a noteworthy challenge in neuroscience. Despite recent progress in neural encoding through CNNs in fMRI studies, significant disparities persist between the computational principles of traditional artificial neurons and actual biological neurons. To tackle this challenge, a framework based on spiking CNNs (SCNNs) for neural encoding was proposed, aiming for greater alignment with biological plausibility [67]. This framework exploits unsupervised SCNNs to extract visual features from image stimuli and utilized a receptive field-based regression algorithm to forecast fMRI responses from these SCNN features. Encoding models were constructed based on SCNNs using four image-fMRI datasets (Figure 3d). Subsequently, image reconstruction and identification tasks were performed using the pre-trained encoding models (Figure 3e,f). Experimental outcomes on handwritten characters, digits, and natural images validate that the projected method achieves notably high encoding performance and can be applied to “brain reading” tasks such as image rebuilding and identification. This study recommended that SNNs hold promise as a valuable approach for neural encoding.

Moreover, a groundbreaking photonic matrix architecture leveraging the real part of a nonuniversal N × N unitary MZI mesh to represent the real-value matrix was proposed by Tian et al. [68]. This innovative approach promises significant advancements, particularly in applications such as PNNs, where it potentially decreases the required MZIs to O(Nlog_2_ N) level while incurring minimal cost to learning capability. In the experimental validation, a 4 × 4 photonic neural chip was successfully realized, and its performance was meticulously assessed in a CNN tasked with handwriting recognition. Remarkably, this 4 × 4 chip demonstrates remarkably low learning capability loss compared to its conventional counterpart, which relies on O(N^2^) MZIs. Furthermore, this architecture showcases superior characteristics across various metrics, including optical loss, chip size, power consumption, and encoding error [68].

### 2.5. Spiking Neural Networks (SNNs)

SNNs signify a novel class of ANNs inspired by the biological neurons’ spiking behavior found in the brain. Unlike traditional ANNs that rely on continuous-valued activations, SNNs communicate through discrete, asynchronous spikes or pulses of activity [17,71]. This spike-based communication enables SNNs to better emulate the dynamics of biological neural systems and potentially achieve higher efficiency in terms of computational resources and energy consumption. In SNNs, neurons integrate incoming spike signals over time, and once a certain threshold is reached, they emit a spike, propagating information to downstream neurons. This temporal aspect of communication allows SNNs to encode data in the specific timing of spikes, enabling them to capture complex temporal patterns and process information more efficiently. SNNs garnered significant interest due to their potential for low-power neuromorphic hardware implementations and their ability to model dynamic spatiotemporal computations, such as sensory processing and event-based vision [72,73]. Despite facing challenges in training and computational complexity, ongoing research into SNNs continues to advance our understanding of neural computation and holds promise for achieving brain-like intelligence in artificial systems [74].

**Figure 4:**
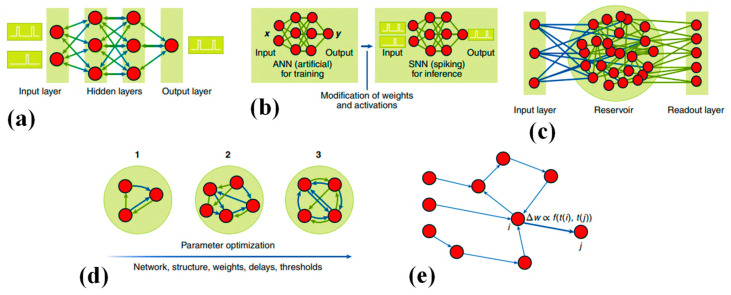
Various training approaches exist for SNNs: (**a**) One approach involves directly training the SNN using spike-based quasi-backpropagation, as illustrated by the network structure depicted [79]. (**b**) Alternatively, traditional ANNs can be trained first and then mapped into SNNs [79]. (**c**) Reservoir computing offers another solution, comprising an input layer, a reservoir, and a readout layer in its typical structure [79]. (**d**) An evolutionary approach involves the gradual evolution of SNN structures and parameters over time [79]. (**e**) Spike-timing-dependent plasticity is characterized by adjusting synaptic weights (Δw) based on the relative spike timings between pre- and post-synaptic neurons [79].

Developing software for a neuromorphic computer often involves designing an SNN tailored for deployment on such hardware. SNNs draw substantial inspiration from biological neural systems, incorporating temporal dynamics into their computation. Within most neuromorphic computers, neurons and synapses in SNNs exhibit time-dependent behaviors. For instance, spiking neurons may gradually lose charge over time according to specific time constants, while SNN elements like neurons and synapses may introduce time delays. The process of crafting algorithms for neuromorphic systems often revolves around defining an SNN suitable for a given application. Various algorithmic strategies exist within neuromorphic computing, broadly categorized into two types: (1) algorithms focused on training or learning an SNN for deployment on a neuromorphic platform (see Figure 4), and (2) non-ML algorithms where SNNs are manually built to address specific tasks. It is important to clarify that in this context, training and learning algorithms pertain to the techniques for enhancing SNN constraints, typically synaptic weights, to tackle a particular problem.

Backpropagation and stochastic gradient descent demonstrated remarkable efficacy in the realm of deep learning. Nonetheless, these methodologies do not directly translate to SNNs due to the nondifferentiable nature of many spiking neuron activation functions, often employing threshold functions. Moreover, the temporal processing aspect of SNNs poses additional challenges in training and learning within these frameworks. Algorithms that excel in deep learning applications require adaptation to operate effectively with SNNs (refer to Figure 4a), with such adjustments potentially compromising the precision of the SNN related to a similar ANN [75]. 

A groundbreaking integrated end-to-end photonic deep neural network (PDNN) designed for sub-nanosecond image classification was presented in [30]. This innovative system operates by directly processing optical waves on an on-chip pixel array as they traverse through layers of neurons. Within each neuron, linear computation occurs optically, while the nonlinear activation function is implemented opto-electronically, resulting in an impressive classification time of under 570 ps, equivalent to a single clock cycle of contemporary digital platforms. The utilization of a homogeneously distributed supply light ensures a consistent per-neuron optical output range, permitting seamless scalability to large-scale PDNNs. Demonstrating remarkable accuracy, the PDNN achieves two-class and four-class classification of handwritten letters with accuracies exceeding 93.8% and 89.8%, respectively [30]. By directly processing optical data without the need for analog-to-digital conversion or large memory modules, this approach promises faster and more energy-efficient neural networks, shaping the next generation of deep learning systems.

Given the established training mechanisms of deep neural networks (DNNs), many efforts towards deploying a neuromorphic solution begin by training a DNN and subsequently converting it to an SNN for inference purposes (see Figure 4b). These approaches have generally yielded performance close to the state-of-the-art, offering significant energy savings by utilizing accumulated computations instead of multiplying and accumulating computations commonly found in DNNs, particularly on datasets like MNIST, CIFAR-10, and ImageNet. Initial conversion techniques often involve weight or activation normalization alongside the use of average pooling instead of max pooling. Some approaches also involve training DNNs under constraints to iteratively shape the neuron’s activation function to resemble that of a spiking neuron. Stockl and colleagues introduced a novel mapping strategy utilizing the Few Spikes neuron model (FS-neuron), capable of temporally representing complex activation functions with at most two spikes. Their method demonstrated near-DNN precisions on standard image classification datasets, meaningfully requiring fewer time steps per inference related to formerly established conversion strategies [76]. Numerous applications showcased on neuromorphic hardware leveraged various mapping techniques discussed above. Tasks such as keyword spotting, medical image analysis, and object detection were proficiently executed on existing platforms such as Intel’s Loihi and IBM’s TrueNorth [77,78].

RC, also known as liquid state machines (refer to Figure 4c), is another prominent algorithm utilized in SNNs. In this approach, a sparse recurrent SNN serves as the “reservoir” or “liquid.” The reservoir, typically randomly configured, must exhibit two critical properties: input separability, ensuring distinct inputs yield distinct outputs, and fading memory, ensuring signals eventually dissipate rather than endlessly propagate through the reservoir. In addition to the untrained reservoir, RC involves a readout mechanism, often implemented as linear regression, which is trained to interpret the reservoir’s output. The main benefit of RC is its elimination of the need to directly train the SNN module. RC in SNNs utilizes sparse and recurrent connections, along with synaptic delays within networks of spiking neurons, to map input into a higher dimensional space, both spatially and temporally. Numerous demonstrations of spike-based RC underscored its effectiveness in processing temporally varying signals. This computing framework comes in various forms, ranging from basic reservoir networks employed in bio-signal processing and prosthetic control applications to more complex architectures, such as hierarchical layers of liquid-state machines. These interconnected layers, often combined with supervised-mode-trained layers, are utilized for tasks involving video and audio signal processing. 

Evolutionary strategies for training or crafting SNNs (refer to Figure 4d) were also employed. Within an evolutionary algorithm, an initial population is established by generating a random array of potential solutions. Each member of this group is assessed and assigned a score, influencing the selection process (favoring superior performers) and reproduction to yield a fresh population. In the domain of SNNs for neuromorphic computing, evolutionary methods can govern various parameters, including neuron thresholds or synaptic delays, as well as the network’s architecture, such as neuron quantity and synaptic connections. These strategies are attractive due to their lack of reliance on activation function differentiability and network structure constraints (e.g., feed-forward vs. recurrent). They also provide the flexibility to evolve both network structure and parameters. However, this adaptability comes with a drawback; evolutionary approaches typically converge more slowly compared to other training techniques. Evolutionary methodologies primarily excelled in control scenarios like video games and autonomous robot navigation.

Numerous neurobiological investigations elucidated the dynamic regulation of synaptic strength driven by the activity of interconnected neurons. This phenomenon was proposed as a fundamental mechanism underlying learning across a spectrum of tasks. Central to this concept is Spike-timing-dependent plasticity (STDP), a pivotal mechanism in the realm of neuromorphic research. STDP operates by fine-tuning synaptic weights according to the precise temporal relationship between spikes from pre- and post-synaptic neurons (See Figure 4e). It stands as one of the most widely utilized synaptic plasticity mechanisms in the burgeoning field of neuromorphic computing, showcasing its significance in mimicking biological learning processes [79].

Several neuroscientific studies delineated the control of synaptic efficacy based on interconnected neuronal activity, proposed as an instrument for learning various tasks. Spike-timing-dependent plasticity (STDP) emerges as the predominant synaptic adaptability mechanism in neuromorphic research, operating by modifying synaptic weights following the timing of spikes between pre- and post-synaptic neurons. Numerous mathematical models of this phenomenon were assessed using datasets such as MNIST, CIFAR-10, and ImageNet. Shrestha et al. proposed a hardware-friendly variant of the exponential STDP rule, although it demonstrated inferior performance in classifying MNIST data compared to the optimal outcomes achieved with SNNs [80]. STDP-inspired principles displayed promise in mimicking diverse ML methodologies such as clustering and Bayesian inference. In applications involving brain–machine interfaces, STDP functions as a clustering mechanism, acting as a sorter for spikes. Moreover, combinations of spiking reservoirs and STDP were incorporated into a framework termed NeuCube, which has been utilized in tasks such as detecting sleep states and controlling prosthetics, processing signals from electroencephalograms, and functional magnetic resonance imaging [81].

### 2.6. Photonic Ising Machines (PIMs)

PIMs represent a revolutionary approach to solving complex optimization problems by leveraging principles from statistical physics and optical computing. Inspired by the Ising model from physics, which describes interactions between spins in a lattice, PIMs utilize networks of interconnected optical components to simulate the behavior of these spins [4]. In PIMs, optical signals represent the spins, and the interactions between them are encoded in the physical properties of light, such as phase or intensity. By exploiting the inherent parallelism and massive computational capacity of light, PIMs can efficiently explore large solution spaces and find optimal configurations for a wide range of optimization tasks [82]. Moreover, PIMs offer benefits in terms of energy efficiency and scalability compared to conventional electronic computing systems. They hold promise for solving combinatorial optimization problems, such as the traveling salesman problem, protein folding, and data clustering, with unprecedented speed and accuracy [82]. While PIMs are still in the early phases of growth, ongoing research and advancements in photonic technologies are driving the realization of practical PIM-based systems, paving the way for transformative applications in various domains, including AI, logistics, finance, and materials science [83].

Ising machines leverage diverse physical systems to efficiently tackle combinatorial optimization problems. Central to their effectiveness is the adaptability of the spin–spin interaction parameter within the Ising model. Choosing an appropriate physical system is crucial for practical machine development. Quantum mechanical phenomena, such as those found in superconducting circuits [84] and trapped ions [85], exploit quantum annealing [86] based on quantum fluctuation. Semiconductor integrated circuits, including CMOS annealing machines [87] and digital annealers [88], emulate simulated annealing (SA). Photonics-based approaches stand out as highly promising for handling large-scale problems due to light’s inherent capabilities for parallel and high-speed processing, along with system robustness. A notable example is the coherent Ising machine [89], employing optical pulses generated by degenerate optical parametric oscillators to implement pseudo-spins [90]. Another innovation is the integrated nanophotonic recurrent Ising sampler, utilizing coherent optical amplitudes for pseudo-spin representation. 

A highly promising method for large-scale light control is spatial light modulation, commonly employed in computing, harnessing light’s parallel propagation traits. An exciting application of this optical technique is the spatial–photonic Ising machine (SPIM) [91], where spins are represented by modulating light waves utilizing a spatial light modulator (SLM). Spin–spin interactions are realized by overlapping light waves through free-space propagation. In comparison to alternative physical implementations, SPIMs offer a simpler configuration and exceptional scalability in spin handling, leveraging light’s parallel propagation based on Fourier optics. These attributes garnered significant attention for SPIMs, leading to the exploration of numerous enhancement avenues. Various approaches, including annealing methods [92], spin encoding techniques [93], interaction models utilizing the transmission matrix of scattering mediums [94], and those exploiting nonlinear optical effects [95], were proposed to advance SPIM capabilities.

**Figure 5:**
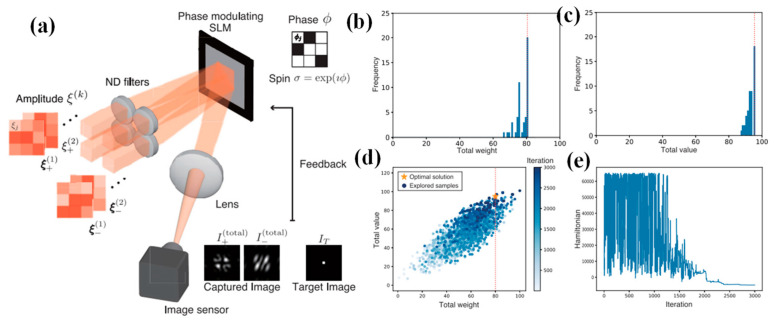
(**a**) Diagram of a SPIM employing physically adjustable SDM-SPIM [96]. Findings from solving the 13-item knapsack problem: (**b**) Distribution of total weight among obtained solutions, illustrated in a histogram [96]. (**c**) Distribution of total value among obtained solutions, depicted in a histogram [96]. (**d**) Demonstration of the distribution of explored specimens [96]. (**e**) Illustration showcasing the time evolution of Ising Hamiltonian values [96].

Sakabe et al. introduce a novel approach, the space-division multiplexed SPIM (SDM-SPIM), offering a versatile system configuration for optically computing the sum of multi-component Hamiltonians while retaining high flexibility in the interaction matrix [96]. The concept of SDM-SPIM is described in Figure 5a. In the SDM scheme, each component’s beams are autonomously controlled to regulate specific optical intensities, enabling the simultaneous physical multiplication of weight coefficients. Consequently, the sum of Ising Hamiltonians for each component is derived by superimposing these beams. Moreover, SDM-SPIM facilitates the physical tuning of optical parameters, including weight coefficients associated with problem constraint conditions, allowing for dynamic optimization processes. This research aimed to authenticate the technique and its capabilities through physical parameter tuning, realized by executing an SPIM with spatial-division multiplexing. A prototype is demonstrated and applied to knapsack problems—a type of combinatorial optimization problem featuring constraint terms. Additionally, the influence of physical parameters is analyzed on this method’s search characteristics and explored techniques to enhance search performance within the SDM-SPIM framework.

Figure 5b,c show histograms displaying the aggregate weight and value of the obtained solutions. These findings unmistakably demonstrate the achievement of the best possible solution, with a total value of 95. Throughout the experimental demonstration, the ultimate solution was identified by selecting the specimen with the uppermost value under the weight constraint from all explored specimens. Figure 5d demonstrates the distributions of specimens attained throughout iterations. The horizontal axis represents the total weight of each specimen, while the vertical axis represents their total value. Notably, with the progression of iterations, the search area gradually converges towards a region surrounding the optimal solution. In Figure 5e, the evolution of the Ising Hamiltonian over iterations is exemplified. After the iteration process, as evident in Figure 5d,e, the near-ground state of the Ising Hamiltonian attained in the experiment unmistakably agrees with specimens resembling the near-optimal solution [96].

### 2.7. Optoelectronic Neural Networks (ONNs)

ONNs represent a convergence of optical and electronic technologies to create powerful and efficient computing systems inspired by the brain’s neural networks [97]. By integrating optical components, such as lasers, photodetectors, and waveguides, with electronic components like transistors and resistors, ONNs harness the strengths of both domains. Optical signals, which travel at the speed of light, enable parallel processing and high-bandwidth communication between neurons, while electronic components provide precise control and computation capabilities. This hybrid architecture allows ONNs to achieve ultra-fast processing speeds and energy-efficient operation, making them well-suited for tasks requiring large-scale parallelism and complex computations, such as pattern recognition, deep learning, and neuromorphic computing. Moreover, ONNs hold promise for addressing challenges in conventional electronic computing systems, including power consumption constraints and interconnect bottlenecks [98]. Ongoing research in optoelectronic materials and device integration is driving the development of increasingly sophisticated and scalable ONN architectures, with potential applications spanning diverse fields such as AI, biomedical engineering, and communication networks. As these technologies continue to advance, ONNs are ready to take on a transformative role in molding the trajectory of computing and information processing [99,100].

ONN represents a capable frontier in AI computing, harnessing parallelization, power efficiency, and speed for advanced applications. Among these, diffractive neural networks stand out, leveraging encoded light transmitted through trained optical modules. Despite their appeal, the expansion of diffractive networks encounters obstacles attributable to the computational and memory demands linked to optical diffraction modeling. To tackle these obstacles, a revolutionary dual-neuron optical artificial learning framework called DANTE is proposed [101]. In DANTE, optical neurons manage the complexities of optical diffraction, while artificial neurons efficiently estimate the demanding optical diffraction computations using lightweight functions. What distinguishes DANTE is its novel convergence strategy, which merges iterative global artificial-learning steps with local optical learning steps. Through rigorous simulation experiments, DANTE achieves unprecedented results, magnificently training large-scale ONNs with 150 million neurons on ImageNet—a milestone earlier thought unfeasible. Furthermore, DANTE notably hastens training paces on the CIFAR-10 benchmark when related to traditional single-neuron learning approaches. In real-world validation, a two-layer ONN system established on DANTE demonstrates its ability to successfully withdraw features and advance the sorting accuracy of natural images. This empirical validation underscores the practical value of DANTE and highlights its potential to propel advancements in ONN technology.

**Figure 6:**
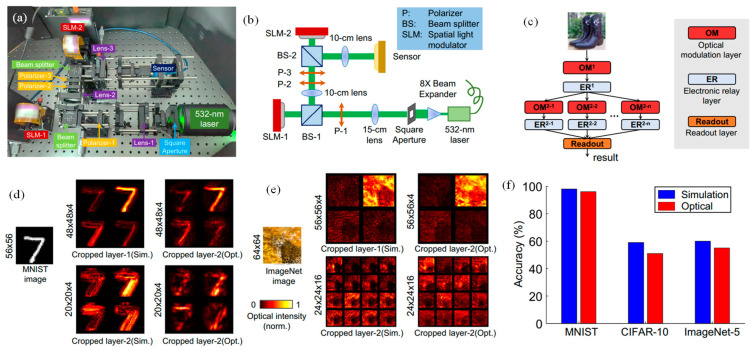
D.A.N.T.E implemented on a physical ONN scheme. (**a**,**b**) The prototype system and its optical setup [101]. (**c**) The network assembly executed in the prototype system [101]. (**d**) Cropped outputs of trained ONN for MNIST classification [101]. (**e**) Outputs of trained ONN for ImageNet classification [101]. (**f**) Analytical and optical accuracy on MNIST, CIFAR-10, and ImageNet datasets [101].

A tailor-made ONN system was devised, harnessing off-the-shelf optical modulation devices to affirm the practical viability of DANTE (illustrated in Figure 6a,b) [101]. This system streamlines the incorporation of optical computing functionalities via a dedicated optical modulation layer. Input signals were modulated through SLM-1, while network parameters underwent modulation via SLM-2, with computing results captured by a CMOS sensor. Additionally, the performance of ONNs was scrutinized using benchmark datasets, including MNIST, CIFAR-10, and ImageNet. The executed two-layer ONN architecture (depicted in Figure 6c) comprised a foundational layer with a solitary optical modulation layer and a subsequent layer with multiple parallel optical modulation layers. The outputs of the second layer were directed to the readout layer to forecast ultimate results [101]. In Figure 6d, MNIST specimen 7 outputs are showcased. The optical intensity maps taken by the sensor closely resemble the simulated outcomes. However, there were discrepancies primarily attributable to imperfect coherent wavefronts and assembly errors in optical modulation devices. To mitigate these errors, the FC layer in the readout layer was re-tuned. In Figure 6e, outputs from the ImageNet-32 dataset, specifically a leopard image, are presented. The variances between simulation and optical results were more pronounced because of the image’s complexity. Nevertheless, similar optical intensity distributions were observed. The optical results appear blurrier, again attributed to assembly errors and system noise. 

Figure 6f presents quantitative analysis results for DANTE. When applied to simple binary-like MNIST datasets, DANTE achieves around 96% accuracy, slightly lower than full simulation results by 2%. The training process involves a global artificial-learning stage that converges in 60 epochs, taking ~2 min 15 s, and a local optical-learning stage requiring around 4 min 40 s to optimize two-phase masks. Retuning the FC layer adds about ½ min, resulting in a total training time of approximately 7 min 25 s. This represents a significant acceleration related to prevailing single-neuron learning approaches such as DPU9, which takes over 5 h for MNIST benchmark training. Looking ahead, integrating the physical ONN system with high-accuracy nanofabrication methods holds promise for meaningfully enhancing its computational capabilities [101].

## 3. Navigating the Landscape of PNNs: Challenges and Prospects

PNNs represent a promising frontier in computing, leveraging the unique properties of light to potentially revolutionize traditional computing architectures [102,103,104]. However, they also face significant challenges that need to be addressed for widespread adoption [105]. One major obstacle is the difficulty of integrating optical components with existing electronic systems, requiring complex and costly hybrid setups. Moreover, the scalability of PNNs remains a challenge, with issues arising from the need for the precise alignment of optical components and limitations in the number of neurons that can be interconnected efficiently [106]. Additionally, noise and signal degradation in optical systems pose significant hurdles to achieving high accuracy and reliability in computation. Despite these challenges, the prospects of PNNs are bright. In our opinion, the advancements in materials science, particularly the creation of innovative photonic materials and nanophotonic devices, offer a hopeful solution to existing constraints, paving the way for more effective and expandable PNNs [107]. Additionally, the natural parallelism and rapidity of optical processing present the opportunity for significant leaps in computational efficiency, especially in areas like pattern recognition and extensive optimization tasks. As ongoing research drives the evolution of optical computing, PNNs stand poised to emerge as a fundamental technology in future computing systems, introducing fresh possibilities and applications across diverse fields [2,108].

The distinctive challenges associated with training PNNs, encompassing the nondifferentiability of numerous spiking neuron activation functions and the temporal processing aspects of SNNs, are indeed notable. These challenges hinder the straightforward application of conventional optimization techniques used in traditional neural networks. However, researchers are actively devising strategies to address these training hurdles. One approach involves developing specialized optimization algorithms tailored for PNN architectures, considering the unique characteristics of photonic devices and spiking neurons [109]. Additionally, advancements in hardware technologies are being explored to enhance the training efficiency of PNNs, such as the integration of neuromorphic computing elements and photonic components optimized for neural network operations [79]. Furthermore, novel approaches that hold promise for streamlining the training process and augmenting the learning capabilities of PNNs are on the horizon. These include the exploration of unsupervised learning methods, reinforcement learning techniques, and leveraging quantum-inspired optimization algorithms to overcome the challenges associated with training PNNs [110]. Overall, ongoing research efforts aim to overcome the inherent training complexities of PNNs and unlock their full potential for a wide range of applications.

Within optical systems, a multitude of factors contribute to performance degradation, particularly notable within PNNs. Thermal noise, generated by random thermal motion within optical components, introduces fluctuations in signal intensity, while shot noise, a consequence of the discrete nature of light, adds inherent randomness to photon arrival times. Additionally, signal attenuation arises from scattering, absorption, and modal dispersion, collectively reducing signal strength and impairing transmission quality over distances. To address these challenges within PNNs, various strategies were proposed. These encompass advanced error correction codes customized for optical communication systems, adaptive signal processing algorithms adept at discerning noise from genuine signals, and innovative optical amplifier designs aimed at amplifying signal strength while mitigating noise interference [111,112,113]. Furthermore, ongoing research explores techniques such as optical phase modulation and dispersion compensation to counteract signal attenuation and distortion, thereby enhancing the resilience and efficacy of optical systems within neural network frameworks [114,115].

The realm of PNN architecture is expansive, encompassing a rich tapestry of techniques and devices utilized to manifest these structures. Within this domain, our exploration unveiled a diverse array of architectures, each leveraging distinct methodologies. Our examination organizes the extensive literature into distinct categories: resonator-based operations, interferometer-based operations, diffractive optics-based operations, and optical amplification/lasing-based operations. Each PNN architecture possesses its unique set of advantages and limitations, and the appropriateness of a specific architecture hinges upon the application context. Resonators, for instance, can store and manipulate light efficiently, facilitating computations. Moreover, their nonlinear behavior is advantageous for implementing activation functions in neural networks. However, resonators are vulnerable to environmental factors like temperature fluctuations and mechanical vibrations, which can adversely impact their performance. Furthermore, the fabrication of high-quality resonators can be both challenging and costly.

Interferometers, on the other hand, excel in manipulating the phase and amplitude of optical signals, thereby enabling complex computations. Their inherent parallel processing capability allows for the simultaneous handling of multiple inputs. Nonetheless, interferometers are susceptible to phase variations and necessitate precise alignment for optimal functionality. Moreover, coherence and stability issues may arise in practical implementations.

Diffractive optics offer the capacity to execute intricate mathematical operations using diffraction patterns. Their parallel computing capability and scalability are notable advantages. However, the introduction of noise and aberrations by diffractive elements can compromise computational accuracy. Additionally, achieving high precision in the fabrication of diffractive optical elements poses a significant challenge.

Optical amplification and lasing mechanisms play a crucial role in amplifying optical signals, facilitating long-distance communication and high-speed processing. Their ability to deliver high gain with low noise characteristics is advantageous. Nonetheless, ensuring stability and preventing instabilities such as mode hopping and noise requires sophisticated control mechanisms. Moreover, the fabrication and integration of optical amplifiers/lasers can be complex and expensive.

In selecting the most suitable PNN architecture for a specific application, various factors must be considered. These include the nature of computations required (e.g., linear vs. nonlinear operations), scalability to handle large-scale neural networks and datasets, robustness against environmental factors and noise, ease of integration with existing photonic or electronic systems, and cost considerations encompassing fabrication, operation, and maintenance.

To advance the implementation of PNNs beyond the existing bulky prototypes built on optical tables, several promising directions can be explored for denser integration. One key approach is the development of integrated photonic circuits, where photonic components are miniaturized and integrated onto a single chip or substrate. This integration could involve leveraging technologies like silicon photonics or photonic integrated circuits (PICs), which enable the creation of complex optical systems on a small footprint [2]. Another direction involves exploring novel materials and structures that can manipulate light at smaller scales, such as metasurfaces or nanophotonic devices. These technologies could allow for the realization of compact and efficient photonic components tailored for neural network applications [116]. Additionally, exploring advanced packaging techniques that facilitate dense stacking and the interconnection of optical elements could lead to more compact and portable PNN implementations. Furthermore, investigating new architectures that optimize the use of light for neural network computations, such as employing reconfigurable photonic networks or hybrid photonic–electronic systems, holds promise for denser and more practical PNN designs. By focusing on these directions, researchers can pave the way for the realization of much denser and scalable PNNs, enabling their integration into a wide range of applications, including efficient deep learning, optical computing, and brain-inspired computing paradigms.

Ultimately, the selection process necessitates a thorough trade-off analysis of these factors to pinpoint the architecture that best aligns with the requirements of the target application. Furthermore, experimental validation and performance evaluation play a pivotal role in assessing the suitability of a particular PNN architecture in practical implementations. For a comprehensive overview, we present a synthesis of the covered architectures in Table 1.

**Figure 7:**
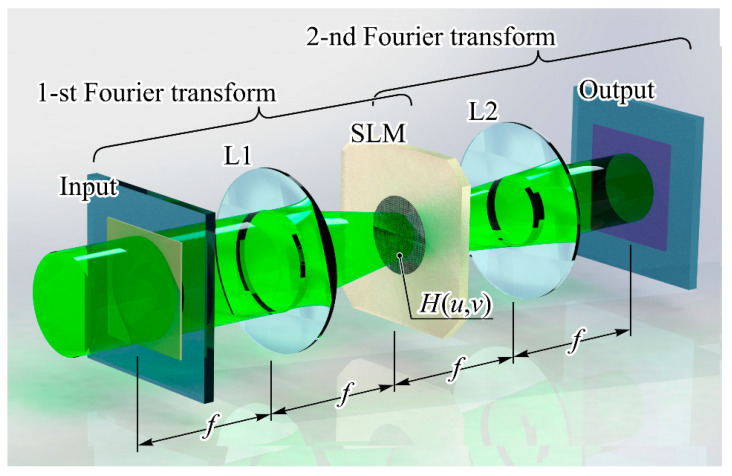
PNN based on the Fourier-correlator scheme (4*f*-system). The mask is determined by the Fourier transform of the kernels from the convolutional layer of a typical CNN.

Furthermore, let us contrast two specific ANN setups by examining their respective mathematical operations. This comparison will enable us to gauge the resulting advantages of optical implementation. The initial configuration is centered around the 4-f Fourier-correlator arrangement (refer to Figure 7), representing an optical CNN with a solitary layer denoted as *H*(*u,v*) [131]. This layer *H*(*u,v*) is defined through the Fourier transformation of the kernel extracted from a standard CNN’s convolutional layer. It can be manifested either as a diffractive optical element (offering damage resistance) or as an SLM (offering dynamic or adaptable properties). The mathematical equivalent to the Fourier-correlator framework comprises two Fourier transformations:

(1) $$W_{f}\left(u,v\right)=\frac{1}{\lambda f}\iint_{R^{2}}w\left(x,y\right)\exp\left[-i\frac{2\pi }{\lambda f}\left(xu+yv\right)\right]dxdy,$$

where *f* is the focal length of a lens and *λ* is the wavelength of the optical radiation and also includes the spatial filtering operation:

(2) $$G(u,v)=W_{f}(u,v)H(u,v).$$

**Figure 8:**
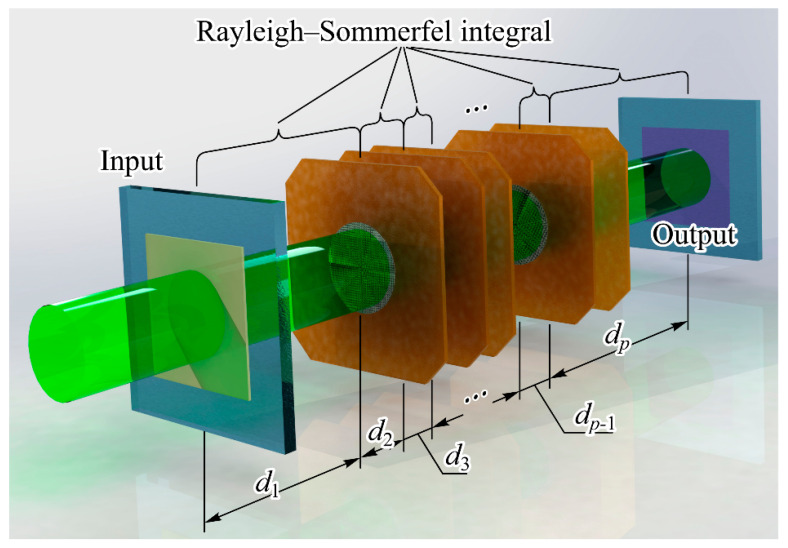
PNN based on the cascade of DOEs, which are pre-trained diffraction layers of a DDNN.

The second configuration (refer to Figure 8) involves a sequence of diffractive optical elements that form pre-trained diffraction layers within a deep diffraction neural network (DDNN) [132]. The mathematical correspondence was established by iteratively applying two operations across successive layers. Specifically, at the *p*th layer, we perform field propagation over a defined distance using the Rayleigh–Sommerfeld diffraction formula [133,134]:

(3) $$w_{p}\left(u,v\right)=-\frac{d_{p}}{2\pi }\iint_{R^{2}}{\hat{w}}_{p-1}\left(x,y\right)\frac{\exp\left(i2\pi R_{p}/\lambda \right)}{R_{p}^{2}}\left(i\frac{2\pi }{\lambda }-\frac{1}{R_{p}}\right)dxdy,$$

where $R_{p}=\sqrt{{\left(x-u\right)}^{2}+{\left(y-v\right)}^{2}+d_{p}^{2}}$ and includes a multiplication of $w_{p}\left(u,v\right)$ by complex values $T_{p}(u,v)$ of the corresponding pre-trained diffraction layer:

(4) $${\hat{w}}_{p}(u,v)=w_{p}(u,v)T_{p}(u,v).$$

Note, in paraxial approximation, one can use the Fresnel–Kirchhoff integral instead of Equation (3):

(5) $$w_{p}(u,v)=-\frac{i\exp\left(i2\pi d_{p}/\lambda \right)}{\lambda d_{p}}\iint_{R^{2}}{\hat{w}}_{p-1}\left(x,y\right){\hat{w}}_{p}(x,y)\exp\left\{\frac{i\pi }{\lambda d_{p}}\left[{\left(x-u\right)}^{2}+{\left(y-v\right)}^{2}\right]\right\}dxdy.$$

The use of Equation (5) can provide the use of some fast calculation algorithms [135]. 

**Figure 9:**
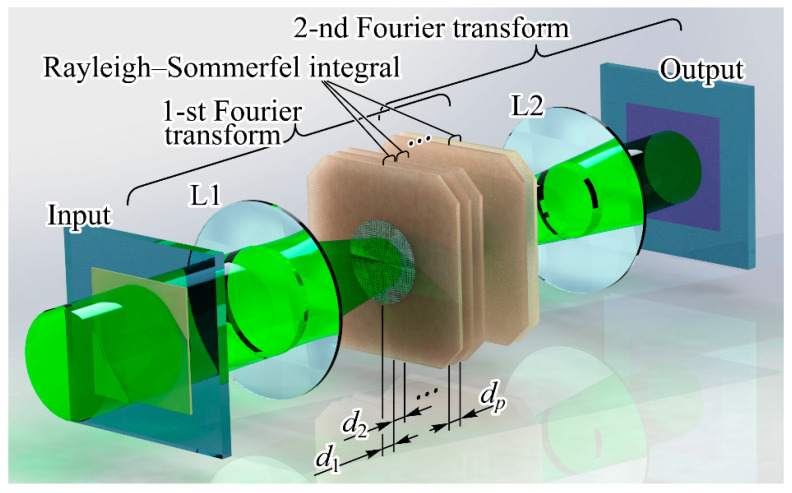
PNN-based on the hybrid of the Fourier-correlator scheme with the cascade of DOEs.

Various composite and hybrid versions of the systems discussed above are possible [136], for example, as in Figure 9.

Considering the fact that the operation of many PNNs is assumed to be closely coupled with a classical computer through input-output devices in the form of SLMs and light-sensitive matrices, it is possible to make a rough estimate of the computing speed in such devices. When calculating, it should be considered that no operations on floating point numbers (Flops) are performed in such systems. I/O devices generate a signal with a bit depth of 1 to 16 bits (most often 8 bits). Therefore, we can talk about either the number of bits per second or operations per second (Ops).

Let us perform a comparative estimation of the speed and efficiency of calculations for PNN using the above two ANN configuration examples (Figure 7 and Figure 8). 

When estimating speed, we can assume that the optical system performs the same mathematical transformations that are used when simulating its operation on a computer. In particular, for the Fourier-correlator scheme (Figure 7), the main computational cost comes from performing two Fourier transforms.

The computational complexity of the discrete Fourier transform (the discrete analog of expression (1)) without the use of fast algorithms is proportional to N4, where N is the dimension of the transformed array. Then, one can write a simple formula to estimate the speed of calculations:

*V* = 2 × *N*^4^ × *r*/*t*, (6)

where *N* is the size of the camera matrix, *t* is the camera exposure time, *r* is the camera bit depth. The speed *V*, in this case, is expressed in bits per second. So, if the SLM with 1024 × 1024 pixels and an operating frequency of 30 Hz is applied with the camera bit depth of 8 bit (approximately corresponds to the physical experiment in [101]), the speed is

*V =* 2.6 × 10^15^ bits/s *≈* 3.2 × 10^14^ bytes/s *=* 3.2 × 10^14^
*Ops =* 320 *TOps,* (7)

The authors of [101] believe that the speed calculation should be based on the fact that the Fourier transform can be calculated using the fast Fourier transform (FFT) and obtain an estimate that is several orders of magnitude lower: *V* = 9 × 10^8^ Ops. However, they point out that the TOps metric may not always accurately reflect actual performance. For example, running two FFTs and one elementwise multiply on an RTX 3090 GPU takes approximately 0.9 ms, which is slower than their PNN prototype (0.7 ms). At the same time, the performance of the RTX 3090, according to the manufacturer, is about 30 TFLops.

In the second scenario, utilizing a PNN built upon a sequence of DOEs (see Figure 8), the significance of estimation increases notably due to the absence of standard rapid algorithms for computing the Rayleigh–Sommerfeld or Kirchhoff integrals. Within discrete analogs of expressions (3) and (5), the most substantial computational challenge lies in evaluating the exponent. Typically, computing the exponent demands approximately two orders of magnitude more operations compared to the simplest binary operation. Therefore, it can be inferred that when computing the Rayleigh–Sommerfeld integral, the number of basic operations will be approximately 100 × N^4^.

If we use SLM with the resolution of 2048 × 2048 pixels and the frequency of 60 Hz as each diffraction element, the number of simple operations on one layer performed in 1 s will be

100 × 2048^4^ × 60 Hz ≈ 10^17^ bits/s ≈ 1.2 × 10^16^ bytes/s = 1.2 × 10^16^*Ops =* 1.2 × 10^4^
*TOps.* (8)

The value (8) is multiplied by the number of layers. It is also quite easy to determine the energy efficiency of calculations for a PNN. In particular, the energy efficiency of 0.02 Tops/W is indicated in [101] for an optoelectronic circuit power of 65 W. However, considering the above calculations, when training a PNN using the Kirchhoff integral, this estimate can be significantly increased up to 100 TOps/W.

## 4. Discussion on PNNs and Concluding Remarks

**Figure 10:**
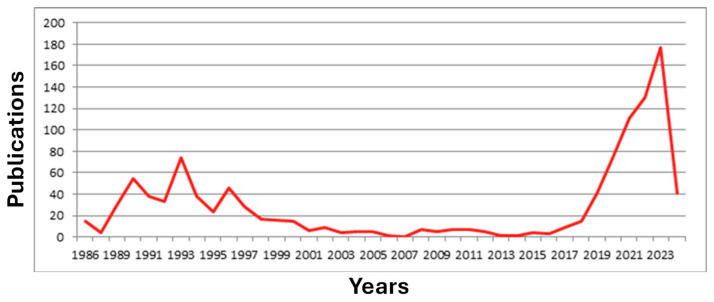
The cumulative number of publications and patents spanning the years 1985 to 2024, focusing on the keywords “optical neural networks” as queried within the Scopus database. These data provide a comprehensive view of the global research landscape surrounding these innovative technologies.

The escalating demand for processing vast amounts of data at ever-increasing speeds has prompted a critical need to surpass the limitations posed by the traditional von Neumann architecture. Innovations in neural network training and testing are imperative, driving the exploration of novel structures capable of accommodating this demand efficiently [137,138]. The surge in research interest is evidenced by the proliferation of publications and patents worldwide, as illustrated in Figure 10. Optical processors, renowned for their exceptional speed and minimal power consumption, emerged as a frontrunner in this pursuit, spurred by the rapid advancements in dedicated hardware for PNNs. Leveraging the inherent advantages of optics, such as high-speed parallel computing and low-energy consumption, optical neural networks exhibit tremendous potential [139]. The trajectory of development in optical neuromorphic computing seems inexorable, with significant strides being made in research and experimentation [103]. Although still in its nascent stages, photonic neuromorphic computing has witnessed the emergence of diverse optimization solutions, indicative of a promising path forward. 

PNNs offer significant advantages over traditional analog neural networks, making them a compelling focus for research and development. One primary benefit is their potential for ultra-fast processing speeds, driven by the high propagation velocity of light compared to electrical signals. This speed advantage can substantially decrease inference times and facilitate the real-time processing of complex data. Additionally, photonic systems inherently support massive parallelism, as light waves can be processed simultaneously across multiple channels, aligning well with the parallel nature of neural network computations. Furthermore, photonic systems are naturally suited for low-power operations, essential for energy-efficient computing. Prioritizing research and development in PNNs over analog alternatives can unlock the unique capabilities of light-based computing, leading to breakthroughs in deep learning, optical computing, and neuromorphic engineering. Leveraging photonics’ advantages may help overcome scalability and speed limitations faced by traditional electronic neural networks, fostering transformative advancements in AI and computational neuroscience.

Designing PNNs often requires specialized software and hardware resources tailored to the unique demands of photonics-based computing. NeuralDesigner is a powerful software tool designed to facilitate the creation, training, and deployment of neural networks for various tasks, including classification, regression, clustering, and forecasting [140]. At its core, NeuralDesigner employs a user-friendly interface that allows users to construct neural network architectures through a visual drag-and-drop approach, eliminating the need for intricate coding. The software employs sophisticated algorithms to automatically optimize network parameters, such as weights and biases, during the training process, thereby enhancing performance and accuracy. Utilizing advanced techniques like backpropagation and gradient descent, NeuralDesigner iteratively refines the network’s parameters based on the provided dataset, ultimately yielding a model capable of making precise predictions or classifications. Furthermore, NeuralDesigner offers comprehensive tools for data preprocessing, validation, and model evaluation, ensuring robustness and reliability in the developed neural networks. With its intuitive interface and robust functionality, NeuralDesigner empowers users to harness the power of neural networks effectively, even without extensive expertise in machine learning algorithms. Additionally, hardware resources like PICs offer a scalable platform for realizing PNNs, leveraging the inherent advantages of photonics, such as high bandwidth, low energy consumption, and parallel processing capabilities. Companies like Lightmatter [141] and Intel’s Silicon Photonics division [142] are at the forefront of developing PICs optimized for PNN applications, providing researchers and engineers with the necessary hardware infrastructure to explore and deploy PNNs effectively.

In the complex architecture of a PNN, the propagation of light signals encounters numerous optical modules, each introducing its own set of challenges. One significant issue is the degradation of light signals as they traverse through these modules due to losses, dispersion, and noise accumulation. To combat this degradation and maintain signal integrity, the network employs a sophisticated mechanism for signal regeneration. This process involves strategically placed regeneration nodes that detect and amplify weakened signals, restoring them to their original strength and clarity. By incorporating such regeneration mechanisms, the PNN ensures that light signals retain their fidelity throughout the intricate pathways of optical processing, facilitating efficient and reliable information transmission and computation. Despite the strides made, challenges persist for PNNs, yet ongoing efforts hold promise for overcoming these hurdles. The comparison of various neural network architectures reveals a spectrum of capabilities and trade-offs, each tailored to specific computational and imaging tasks [9].

The training process of PNNs diverges significantly from that of ANNs. Unlike ANNs, which rely on electronic signals for computation, PNNs leverage photons, the fundamental particles of light, to perform calculations. PNNs encode data into optical signals, which propagate through photonic circuits that emulate the functionalities of neurons and synapses. This optical computing paradigm offers unique advantages such as high parallelism, low energy consumption, and potentially high-speed processing due to the intrinsic speed of light [143]. However, the training of PNNs can be more challenging compared to ANNs due to the specialized hardware requirements and the complexity of optical signal processing. While PNNs hold promise for ultra-fast computation and the efficient processing of massive datasets, their training speed may not necessarily surpass that of ANNs in all scenarios. The optimization and calibration of photonic components and the development of suitable training algorithms are ongoing areas of research aimed at enhancing the efficiency and scalability of PNN training [144]. Therefore, while PNNs offer exciting prospects for future computing paradigms, their training speed and effectiveness currently depend on various factors and remain an active area of investigation in the field of photonics and neuromorphic computing [2].

FNNs offer simplicity and efficiency, making them suitable for structured data analysis and pattern recognition [145,146]. FNNs have a multitude of potential implications across various domains. In finance, they can be employed for stock market prediction and algorithmic trading [147,148]. In healthcare, they may aid in disease diagnosis and drug discovery. Within the realm of autonomous vehicles, FNNs can contribute to advanced driver assistance systems and collision avoidance. Moreover, in natural language processing, they can enhance language translation and sentiment analysis. Overall, the potential implications of FNNs span across industries, promising advancements in efficiency, accuracy, and decision-making processes.

RC is a novel approach to ML that harnesses the dynamics of a fixed RNN known as the reservoir. This network is untrained, acting as a reservoir of dynamics to process input signals [149]. The output layer, which is trained to perform a specific task, reads the state of the reservoir to generate predictions or classifications. The potential implications of RC are vast. In fields like time series prediction, RC offers remarkable accuracy and efficiency, outperforming traditional methods. It also shows promise in areas such as speech recognition, where its ability to capture temporal dependencies leads to improved performance. Moreover, RC has applications in robotics, control systems, and cognitive modeling, suggesting its potential for advancing various domains of AI [150,151]. Its efficient training process and adaptability make RC a promising avenue for tackling complex real-world problems.

CNNs, renowned for their hierarchical feature extraction and translational invariance, dominate image-related tasks with their ability to capture spatial hierarchies effectively [152]. The effectiveness of classification, measured by metrics like accuracy, misclassification rate, precision, and recall, is significantly influenced by the configuration of convolutional layers in a CNN. Factors such as the number of pooling layers, filters, filter sizes, stride rates, and pooling layer placements play pivotal roles in shaping CNN performance. Given the resource-intensive nature of CNN training, reliant on potent hardware like GPUs, extensive experimentation with different parameter combinations demands substantial time and computational resources [153]. Hyper-parameter selection profoundly impacts CNN performance, with even minor adjustments capable of yielding significant changes. Therefore, meticulous consideration of parameter choices is imperative when devising optimization strategies. Over time, CNN architectures have evolved from modest layer counts (e.g., AlexNet) to encompass hundreds of layers, thereby enhancing compactness and effectiveness (e.g., ResNet, ResNext, DenseNet). However, these advancements introduce immense model complexities, necessitating large datasets and powerful GPUs for training. Consequently, there is a burgeoning interest in developing lightweight networks to mitigate redundancy further. Choosing the optimal detection network for a specific application and embedded hardware entails striking a balance between speed, memory utilization, and accuracy. Preferably, compact models with fewer parameters should be prioritized, even if it entails sacrificing detection accuracy initially [154]. Techniques like hint learning, knowledge distillation, and refined pre-training methods offer avenues for compensating for this reduction in accuracy. These enhancements empower CNNs to glean insights from data at varying depths and structural configurations. Recent studies advocate for utilizing blocks instead of conventional layers, showcasing considerable potential for enhancing CNN performance.

RNNs offer several advantages in the realm of sequential data processing [155]. One significant advantage is their ability to capture and utilize temporal dependencies within sequential data, making them well-suited for tasks such as time series prediction, natural language processing, and speech recognition. Furthermore, RNNs are highly flexible and adaptable, capable of processing sequences of varying lengths, which is crucial for handling real-world data with irregular temporal structures. Additionally, advancements such as Long Short-Term Memory (LSTM) and Gated Recurrent Unit (GRU) architectures address the vanishing gradient problem associated with traditional RNNs, enhancing their ability to capture long-term dependencies and improving overall performance on complex sequential tasks [156].

SNNs, inspired by biological neurons, excel in energy efficiency and event-driven computation, making them ideal for neuromorphic computing and real-time applications. SNNs offer promising applications due to their resemblance to biological brains and unique computational capabilities [157]. Particularly in neuromorphic engineering, SNNs closely mimic biological brain functions, making them ideal for tasks like sensory processing, pattern recognition, and motor control in robots and autonomous systems [158,159]. Their event-driven nature also makes them advantageous for low-power computing environments, making them suitable for IoT sensors and wearable electronics. Moreover, SNNs contribute to neuroscience research by modeling neural dynamics and enhancing the understanding of brain functions, thereby advancing cognitive science and brain–computer interfaces [160]. 

PIMs harness the power of optics for parallel processing and optimization tasks, potentially outperforming conventional computing for specific problems [83]. Utilizing principles from both quantum and classical physics enables the tackling of mathematical computations that pose challenges to conventional electronics. Recently, PIMs have emerged, showcasing the ability to compute spin Hamiltonian minima, offering a pathway to groundbreaking hardware for accelerated ML [91]. However, existing systems face scalability issues or are constrained by a limited number of spins. In response, a large-scale optical Ising machine using a straightforward setup with a spatial light modulator was demonstrated. The experiments achieve configurations encompassing thousands of spins, converging to ground states within a low-temperature ferromagnetic-like phase, featuring all-to-all and adjustable pairwise interactions. These findings pave the way for classical and quantum PIMs, harnessing light’s spatial degrees of freedom for parallel processing of extensive spin systems with programmable couplings [91].

ONNs blend optics and electronics, offering high-speed processing and massive parallelism, albeit with challenges in hardware complexity and fabrication costs [99,161]. ONNs offer vast potential across diverse applications thanks to their unique integration of optics and electronics, which grants them advantages in speed, energy efficiency, and parallel processing capabilities. A notable application lies within AI and ML tasks, where these networks excel in accelerating intricate computations, facilitating the real-time analysis of extensive datasets. This capability proves invaluable in domains such as image and pattern recognition, natural language processing, and autonomous systems [162]. Furthermore, ONNs exhibit promise in areas like medical diagnostics, where the swift and precise analysis of medical imaging data is essential for timely diagnosis and treatment planning. Leveraging their parallel processing capability enhances the speed and accuracy of interpreting medical images, thereby improving healthcare outcomes [163,164]. Moreover, these networks hold potential in communication and data processing networks, offering high bandwidth and low power consumption, thereby enhancing the efficiency of data transmission and processing. This contributes to the development of faster and more energy-efficient communication systems [165]. In computing and imaging, the choice among these architectures depends on factors such as data characteristics, computational requirements, and performance objectives. While each architecture presents unique strengths and weaknesses, ongoing research and technological advancements continue to refine these models, promising further innovations in neural network computing and imaging applications and shaping the future of AI-driven solutions across diverse domains.

In the end, we would like to conclude the paper by stating that there are still several hurdles that must be overcome for PNNs to become practical, even for niche applications. One significant challenge is the development of efficient and compact photonic components that can perform neural network operations reliably. Current photonic devices often rely on bulky and expensive setups, requiring precise alignment and stabilization, which limits their practical deployment. Miniaturizing and integrating these components into scalable systems, such as PICs, is essential for practical PNN implementation. Another obstacle is achieving compatibility between photonic and electronic systems for data interfacing and processing, as seamless integration with existing computing platforms is crucial for adoption. Additionally, addressing issues related to noise, nonlinearities, and signal loss in photonic systems is essential to ensure the accuracy and robustness of PNNs. Moreover, developing efficient training algorithms specifically tailored for photonic hardware and exploring novel architectures optimized for light-based computations are critical steps toward practical PNNs. Overcoming these hurdles will unlock the full potential of PNNs, enabling their use in diverse applications ranging from high-speed computing to neuromorphic engineering and beyond.

## Figures and Tables

**Table 1:** Compilation of several noteworthy previous studies on PNN Structure.

Devices	Application	Results	Ref.
Micro-rings	Lorenz attractor simulation to benchmark against a traditional CPU-based continuous time RNN	Reports 294× acceleration in simulation over traditional CPU-based continuous time RNN	[117]
VSCEL-Sas	SNN for learning and recognizing arbitrary spike patterns	-	[118]
SOA-MZIs	RNN benchmarked using a finance forecasting application utilizing the FI-2010 dataset	Gated optical RNN achieved an F1 score of 41.85%	[119]
Micro-rings and SOAs.	Various benchmarks, including MNIST, tested on photonic CNNs	Reduction in operation cost when compared to GPU-based implementations, with up to 25× better computational efficiency	[120]
Micro-rings	MNIST classification using CNNs	Faster when compared to GPU-based implementations and 0.75 times the power consumption	[121]
Micro-disks	Binarized CNN acceleration for MNIST and ImageNet classification	16.9× better FPS and 17.5× FPS/W over [122]	[123]
VCSOA and VCSELs	SNN for learning and recognizing arbitrary spike	-	[124]
SOAs and AWGs	DNN implementation. Tested on Fisher’s Iris classification	A prediction accuracy of 85.8% achieved	[125]
SOA	Spoken digit recognition using RC	The work reports a minimum WER of 4.5% for their coherent SOA-based reservoir	[126]
MZIs	Photonic DNN for vowel recognition	Achieved 76.7% accuracy in vowel recognition	[127]
MZIs	MNIST dataset classification using structured NN	98.5% accuracy	[128]
MRs	This exercise aimed to demonstrate the viability of B&W-based SNNs. No experiments based on applications were carried out in this study.	-	[129]
GST-embedded MRs	MNIST classification with MLPs	98.06% accuracy	[130]

## Data Availability

Not applicable.
